# Enhanced expression of histone chaperone APLF associate with breast cancer

**DOI:** 10.1186/s12943-018-0826-9

**Published:** 2018-03-26

**Authors:** Aditi Majumder, Khaja Moheiddin Syed, Ananda Mukherjee, Manendra Babu Lankadasari, Juberiya Mohammed Azeez, Sreeharshan Sreeja, Kuzhuvelil B. Harikumar, Madhavan Radhakrishna Pillai, Debasree Dutta

**Affiliations:** Rajiv Gandhi Centre for Biotechnology, Cancer Research Program, Thycaud PO, Poojappura, Thiruvananthapuram, 695014 India

**Keywords:** Histone chaperone, APLF, Invasive breast cancer, EMT

## Abstract

DNA damage-specific histone chaperone Aprataxin PNK-like factor (APLF) regulates mesenchymal-to-epithelial transition (MET) during cellular reprogramming. We investigated the role of APLF in epithelial-to-mesenchymal transition (EMT) linked to breast cancer invasiveness and metastasis. Here, we show that a significant manifestation of APLF is present in tumor sections of patients with invasive ductal carcinoma when compared to their normal adjacent tissues. APLF was significantly induced in triple negative breast cancer (TNBC) cells, MDAMB-231, in comparison to invasive MCF7 or normal MCF10A breast cells and supported by studies on invasive breast carcinoma in The Cancer Genome Atlas (TCGA). Functionally, APLF downregulation inhibited proliferative capacity, altered cell cycle behavior, induced apoptosis and impaired DNA repair ability of MDAMB-231 cells. Reduction in APLF level impeded invasive, migratory, tumorigenic and metastatic potential of TNBC cells with loss in expression of genes associated with EMT while upregulation of MET-specific gene *E-cadherin* (*CDH1*). So, here we provided novel evidence for enrichment of APLF in breast tumors, which could regulate metastasis-associated EMT in invasive breast cancer. We anticipate that APLF could be exploited as a biomarker for breast tumors and additionally could be targeted in sensitizing cancer cells towards DNA damaging agents.

**Electronic supplementary material:**

The online version of this article (10.1186/s12943-018-0826-9) contains supplementary material, which is available to authorized users.

Recently, we demonstrated that reduced expression of histone chaperone APLF could enhance the kinetics and efficiency of reprogramming of mouse embryonic fibroblasts to induced pluripotent stem cells [1]. APLF act as a DNA repair factor involved in non- homologous end joining (NHEJ) mediated repair of DNA double strand breaks (DSBs) [2]. As a histone chaperone, APLF specifically bind to histones H3/H4 tetramer and could recruit variants of histone H2A at the damaged sites on DNA [3]. We demonstrated that APLF downregulation augmented mesenchymal-to-epithelial transition (MET) associated with cellular reprogramming of mouse embryonic fibroblasts [1]. Thus, we hypothesized that APLF could induce the reverse phenomenon, epithelial-to-mesenchymal transition (EMT). Being a salient feature of development and wound healing, EMT additionally attributes to the invasive and metastatic behavior of tumor cells. So, based on the involvement of APLF in MET and DNA repair, we studied the role of APLF in breast cancer.

## Results and discussions

### Enhanced expression of APLF in breast cancer

**Fig. 1:**
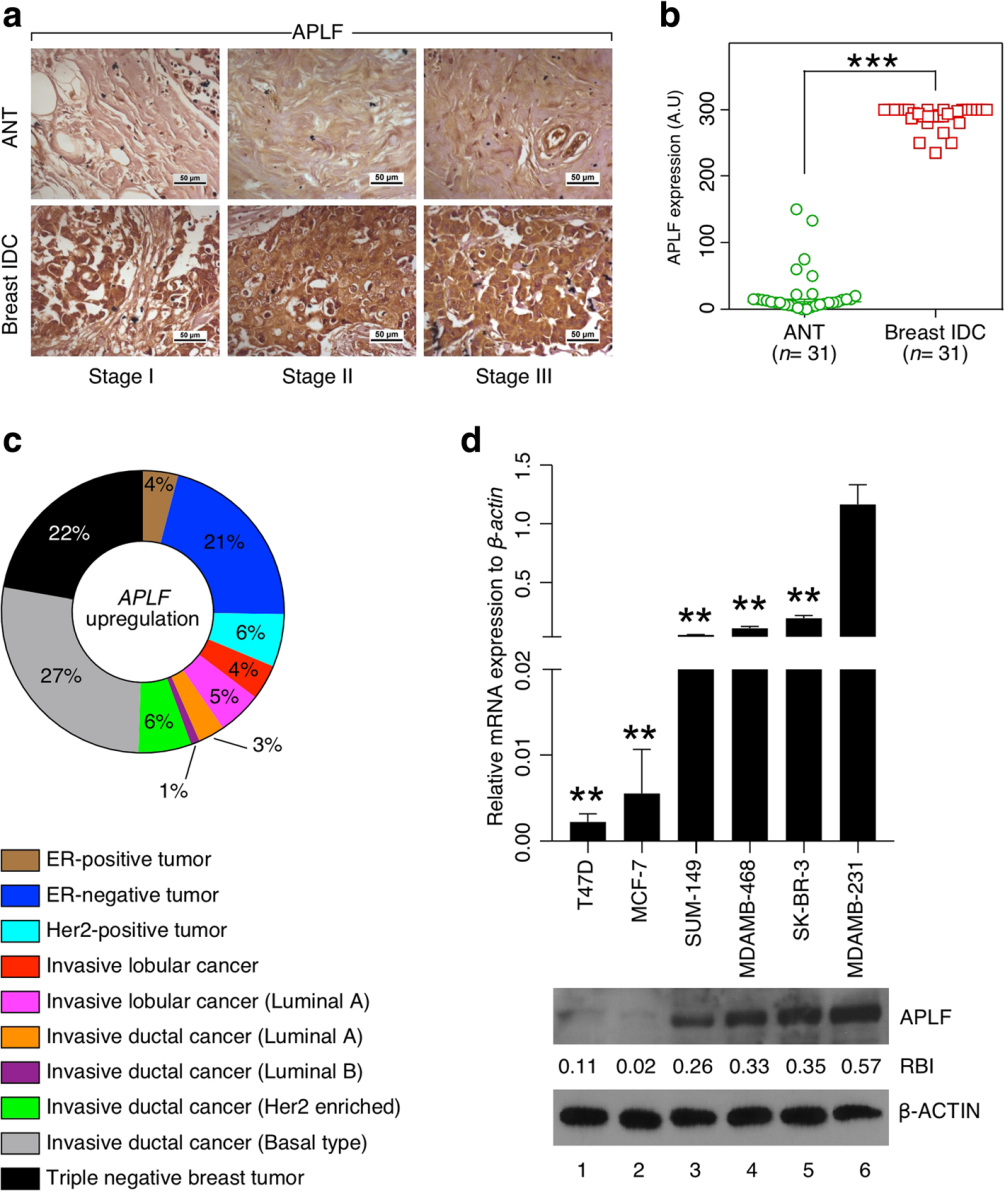
Enhanced APLF expression mark breast cancer. **a** Representative picture from tissue array (US biomax Inc., #OD-CT-RpBre03-004) for invasive ductal breast carcinoma (IDC) with 31 matched control and normal (adjacent tissue or ANT) sections were investigated for the expression of APLF by IHC following standard protocol. Scale bar: 50 μm. **b** Plots represent expression of APLF in adjacent normal tissues (ANT) and matched tumors respectively. Expression values were determined by histo-scoring and expressed as median with 95% confidence interval. Statistical significance was determined using Wilcoxon rank sum test. A.U. = Arbitrary Unit. **c** TCGA study on invasive breast carcinoma samples was analyzed. Pie chart represents the percentage of alteration in *APLF* mRNA expression among different subtypes of breast cancer patients [5]. **d** Different breast cancer cell lines were considered for the expression of APLF with varying degree of invasive potential. mRNA and protein were isolated from all these cell lines and observed for the expression of APLF by qRT-PCR (upper panel) and western blot analysis (lower panel). Error bar = S.E.M for three independent experiments. Statistical analysis was performed using Student t-Test function, *p < 0.05, **p < 0.01. Band intensity was measured by ImageJ software, RBI = Relative Band Intensity. A representative image for the blot has been presented

Tissue array (US Biomax, Inc., #OD-CT-RpBre03-004) for invasive ductal breast carcinoma with 31 matched control and normal (adjacent tissue or ANT) sections were investigated for the expression of APLF. Tumor sections demonstrated significant enrichment in APLF expression compared to matched normal sections (Fig. 1a, b). To ensure the uniformity of the results, we acquired invasive ductal breast tumors and ANTs from Regional Cancer Centre, Thiruvananthapuram, India and performed IHC with another APLF antibody (indicated as #2), generated in Prof. Ivan Ahel lab at Oxford University (a kind gift) [3] along with the commercially available antibody (antibody #1). A similar trend in APLF appearance in the tumor vs. normal adjacent section was observed irrespective of the source of antibody or patient sample (Additional file 1: Figure S1A). Immunofluorescence study on APLF expression in the aforementioned samples demonstrated significantly enhanced expression of APLF in the tumor section (Additional file 1: Figure S1B). Analysis of TCGA study on invasive breast carcinoma [4] for *APLF* alterations, comprising of a large cohort of patient samples (*n* = 817), further demonstrated an upregulation of *APLF* mRNA in invasive ductal carcinoma (IDC) of basal origin (27%) and in patients with triple negative breast tumors (22%) (Fig. 1c). Thus, TCGA study demonstrated substantial increase in APLF level with increase in invasive behavior of the breast cancer subtypes. Next, we determined APLF level among cell lines with varying degree of invasive and metastatic potential. Normal breast cells MCF10A expressed minimal level of APLF followed by invasive MCF7 cells and the highest in TNBC MDAMB-231 cells, both being derived from patients diagnosed with IDC (Additional file 1: Figure S2A). Significantly lower level of APLF was expressed in pure luminal subtype MCF7 and T47D than that of cell lines of basal origin including MDAMB-468, SUM149 and MDAMB-231 (Fig. 1d). Migration and invasive potential of MCF7 was least, followed by SKBR3 and the maximum in MDAMB-231 cells (Additional file 1: Figure S2B, S2C). Increase in APLF expression in MDAMB-231 cells was further confirmed by the use of APLF antibody#2 (Additional file 1: Figure S2D). We were intrigued to understand whether this is a general phenomenon prevalent in any cancer or restricted to breast cancer only? So, we analyzed Cancer Cell Line Encyclopedia for *APLF* expression in invasive and metastatic cell lines that originated from different primary adenocarcinomas [5]. Interestingly, reduced expression of *APLF* was observed in cell lines from pancreas, large intestine and prostate unlike induced expression in MDAMB-231 cells (Additional file 1: Figure S2E). In syngeneic colon cancer cell lines, SW480 and SW620, no significant change in APLF expression was observed upon consideration of their different metastatic potential (Additional file 1: Figure S2F). Thus, tissue array of patient samples, cell lines and analyses of TCGA studies provided evidence that APLF upregulation associate with breast cancer and thus warrant an understanding on the role of APLF in breast cancer.

### APLF regulate cellular machinery

**Fig. 2:**
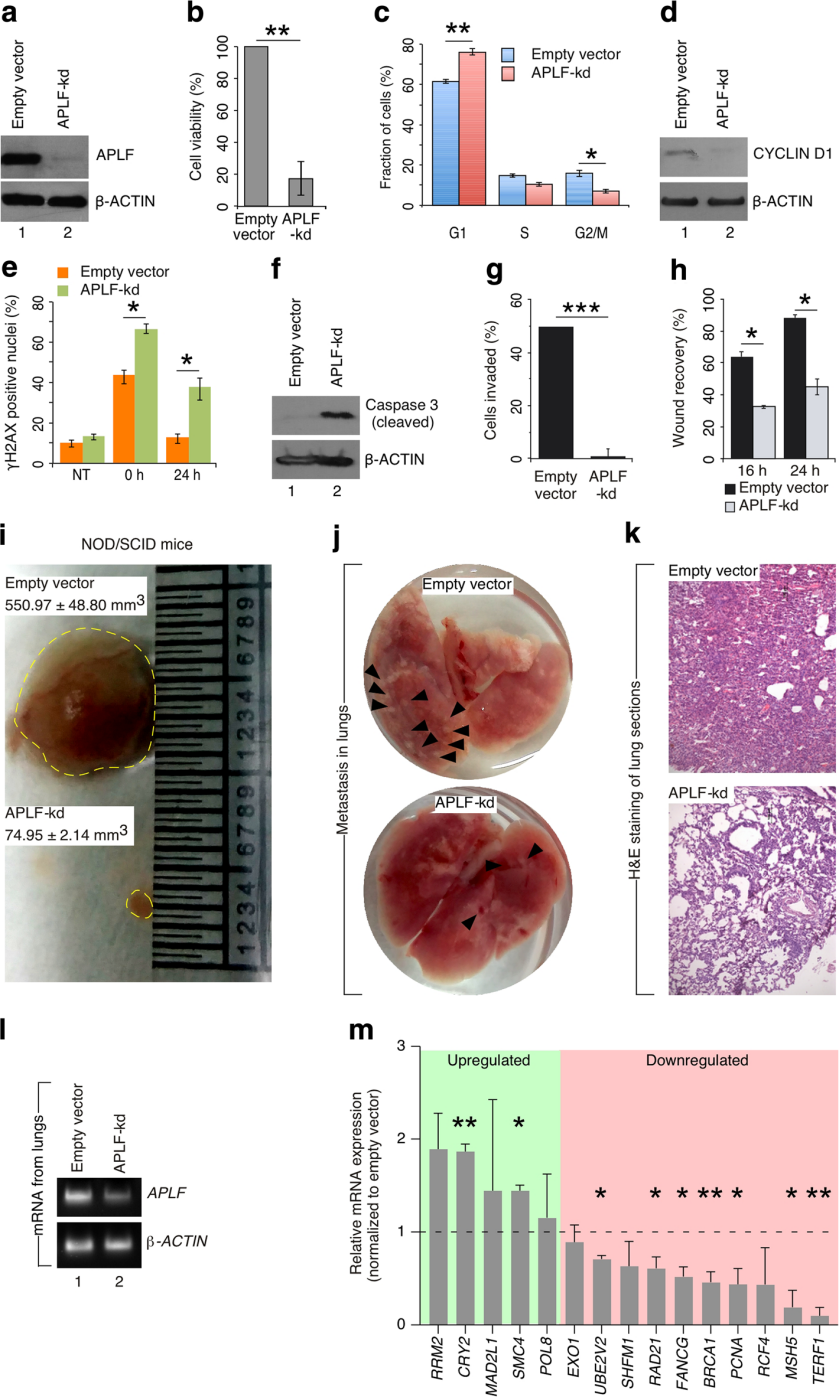
APLF downregulation influence cellular machinery and impede invasive, tumorigenic and metastatic potential of metastatic MDAMB-231 cells. **a** MDAMB-231 cells were transduced with lentiviral particles expressing shRNA against *APLF* or empty pLKO.1 vector (empty vector). Lentiviral vectors containing shRNA targeting human *APLF* was cloned in the pLKO.1 (Addgene) vector [1]. Extent of knockdown was measured at the protein level by western blot. **b** Viability of the control and *APLF*-kd MDAMB-231 cells were determined by MTT assay. **c** Cell cycle analysis for both control and *APLF*-kd cells were performed. Representative plot indicate the percentage of cells present in a given phase for control and *APLF*-kd cells. **d** G1/S-phase specific marker, CYCLIN D1 level was determined in control and *APLF*-kd cells by western blot analysis. **e** Control and *APLF*-kd cells were exposed to DNA DSB inducing agent etoposide (10 μM) for 4 h followed by recovery in absence of etoposide. γH2AX-positive foci cells were determined by immunofluorescence analysis to demonstrate the defect in DNA repairs after 0 h and 24 h of recovery period. Bar graph representing the fraction of γH2AX–positive foci in control and *APLF*-kd cells. Nuclei with ≥5 foci were counted as positive. **f** Same set of samples was analyzed for the expression of cleaved Caspase 3 by western blot as a measure of apoptosis in response to APLF-knockdown. **g** Invasion assay was performed in invasion chamber from Corning (Corning® BioCoat™ Matrigel® Invasion Chamber; 354,480). The graph represent the percentage of cells invaded and expressed in terms number of cells invaded to total number of cells added to the upper chamber at the start of the experiment. **h** Same set of cells were investigated for their migration or wound healing potential. Bar graph represents percentage of wound recovery expressed in terms of [1-(Width of the wound at a given time/width of the wound at *t* = 0)] for control and *APLF*-kd MDAMB-231 cells. **i** Control and *APLF*-kd MDAMB-231 cells were subcutaneously injected in female NOD/SCID mice (*n* = 3 for each group; age = 6-8 weeks). After 5 weeks, mice injected with control cells developed tumors of significantly bigger size than in mice injected with *APLF*-kd cells. Representative picture has been included and the experiment was repeated independently 3 times. **j**, **k** To determine the effect of APLF on in vivo metastatic potential, both control and *APLF*-kd cells were injected into the lateral tail vein of female NOD/SCID mice (*n* = 3 for each group; age = 6-8 weeks). Prior to this, control and *APLF*-kd MDAMB were transfected with pEGFPC1 (Clonetech; 6084-1). After 6 weeks of injection, lungs were dissected and examined for the presence of metastatic nodules (black arrows). Representative lung and H&E staining of metastatic tumor are shown. **l** Expression of *APLF* in lungs was determined by RT-PCR. Human *APLF* and *ACTIN* confirmed the presence of MDAMB-231 cells in the lungs section. Mouse *Gapdh* was used as the negative control. **m** Control and *APLF*-kd MDAMB-231 cells were investigated for the expression of DNA repair genes associated with breast cancer metastasis. mRNA was extracted and analyzed for the expression of genes by qRT-PCR. Error bar = S.E.M for three independent experiments. Statistical analyses were performed using Student t-Test function, *p < 0.05, **p < 0.01

Often, proliferation potential of cancer cells has been correlated to the aggressiveness of the tumor. As APLF expression is highest in MDAMB-231 cells, we exploited them for further mechanistic studies. To understand the role of enhanced expression of APLF, we stably downregulated APLF expression by shRNA mediated knockdown (mentioned as APLF*-*kd hereafter). A significant depletion in APLF expression to 90% was observed in MDAMB-231 cells (Fig. 2a). In silico analysis for checking shRNA specificity using SpliceCenter [http://projects.insilico.us/SpliceCenter/siRNACheck], web-based bioinformatics tool, could not detect any off-targets for this *APLF* shRNA (Additional file 1: Figure S3A). An additional assay was performed to check the specificity of the APLF antibody. HEK293 cells, which has very low to almost undetectable endogenous level of APLF, upon ectopic expression, showed significant increase in APLF level and thus proved the specificity of the antibody (Additional file 1: Figure S3B, S3C). APLF-downregulation decreased the survivability of MDAMB-231 cells to 20% of control cells (Fig. 2b). Cell cycle analysis, after synchronization at G0/G1 phase by serum starvation, demonstrated significant increase in G1-phase population of APLF-kd cells whereas reduction in S and G2/M phase population compared to control cells (Fig. 2c). But, is this effect of APLF restricted to tumor-specific cells or is a global phenomenon? To answer that, we knocked down the APLF expression in MCF10A, normal breast cell line (Additional file 1: Figure S4A). Downregulation of APLF could not alter the distribution of MCF10A cell population among different phases of cell cycle when compared to the control cells (Additional file 1: Figure S4B). Phenotypically, APLF-kd cells were indistinguishable from the control MCF10A cells (Additional file 1: Figure S4C). Thus, effect of APLF downregulation is tumor-specific and not global.

Ectopic expression of *APLF* in MDAMB-231 cells (Additional file 1: Figure S4D, S4E) resulted in increased S-phase and G2/M-phase population while reduction in G1-phase population compared to control MDAMB-231 cells (Additional file 1: Figure S4F). CyclinD1, associated with increased rate of proliferation, marks G1-S phase transition and APLF-downregulation resulted in reduced CYCLIN D1 level in MDAMB-231 cells (Fig. 2d). Interestingly, histone variant MacroH2A.1, a repressive chromatin mark, could regulate the Cyclin D1 expression in different types of cancer including melanoma and osteosarcoma. APLF could recruit MacroH2A.1 at DNA damage site [3] as well as within the promoters of transcription factor associated with EMT [1]. Upon downregulation of APLF, significant enrichment of MACROH2A.1 was observed at the *CYCLIN D1* promoter in APLF-kd cells in comparison to control cells (Additional file 1: Figure S4G). This recruitment could lead to the repression in Cyclin D1 expression resulting in inhibition in proliferation of APLF-kd MDAMB-231 cells.

*APLF*^*−/−*^ mice have a mildly attenuated DNA repair defect and could delay the development of myeloid neoplasm upon exposure to ionizing radiation. In response to DNA-double strand break inducing agent etoposide, at 0 h post-recovery, γH2AX accumulation significantly increased in APLF-kd cells than in control cells (Fig. 2e). With time, γH2AX level reduced and achieved a similar level to untreated control cells at 24 h, whereas APLF-kd cells retained ~ 40% of γH2AX level even after 24 h of recovery (Fig. 2e). So, downregulation of APLF could compromise damage-induced DNA repair machinery thereby enhancing the probability in cell death arising from inefficient DNA damage response. APLF is a constituent of the NHEJ repair pathway. Along with its interacting partner PARP3, APLF accelerates the NHEJ by providing a scaffold for the recruitment of Ku complexes [2]. So, a defect in the DNA repair is not unexpected in cells with reduced level of APLF.

Loss in survivability in response to APLF downregulation in MDAMB-231 cells might be the manifestation in cell death by apoptosis or necrosis. Annexin-V and PI staining demonstrated significant increase in apoptosis in APLF-kd MDAMB-231 cells (population doubled in APLF-kd cells to control cells) (Additional file 1: Figure S4H). This was further confirmed by induced expression of cleaved Caspase 3, the primary apoptotic factor associated with DNA fragmentation (Fig. 2f).

To demonstrate the specificity of the shRNA, *APLF* shRNA-resistant MDAMB-231 cells were analyzed for the effect of APLF-knockdown. Cell survivability was significantly increased in *APLF*-shRNA-resistant cells in comparison to APLF-kd cells (Additional file 1: S5A, S5B). Distribution of cell population among the different phases in *APLF*-shRNA-resistant cells was similar to the control MDAMB-231 cells (Fig. 2c). So, functionally APLF could modulate the entry of cells into cell cycle and interfere with the proliferative capacity of the breast cancer cells.

### APLF modulate invasive, tumorigenic and metastatic potential

To determine the role of APLF in invasiveness, we performed an in vitro matrigel invasion assay. Only 2% of *APLF*-kd cells could invade the membrane in comparison to 50% of control MDAMB-231 cells (Fig. 2g). Prior to this, we detected whether there is any significant loss in cell number due to apoptosis in APLF-kd MDAMB-231 cells during the time period necessary to perform the invasion assay. Around 40 h of continuous culture did not induce apoptosis in APLF-kd cells in comparison to control cells (Additional file 1: Figure S6A). Ectopic expression of *APLF* (Additional file 1: Figure S4D, S4E), enhanced the invading potential to 2-fold compared to control cells (Additional file 1: Figure S6B). Cell migration, measured by wound closure assay, was significantly reduced in *APLF*-kd cells to control cells (Fig. 2h). Upon subcutaneous injection of control and *APLF*-kd cells into immune-compromised mice, tumor generated from APLF-kd cells was significantly reduced in size than the tumors generated from control cells (Fig. 2i). This was expected due to severe loss in cell survivability, cell cycle arrest and increased apoptosis upon downregulation of APLF in MDAMB-231 cells. Next, we evaluated the effect of APLF depletion on metastasis. Upon lateral tail vein injection of control and APLF-kd MDAMB-231 cells into immune-compromised mice, significant increase in number of metastatic nodules in the lungs were observed in mice injected with control cells (Fig. 2j, k). mRNA analysis demonstrated significant reduction in the APLF level within the lung sections derived from mice injected with *APLF*-kd cells (Fig. 2l). Thus, APLF-downregulation impeded invasive, tumorigenic and metastatic potential of TNBC, MDAMB-231 cells. Although cell proliferation, tumor growth or invasion/migration potential are all distinct features of carcinogenesis, but what we could infer from the results demonstrated here that cell cycle arrest resulting due to APLF downregulation in MDAMB-231 cells might be the primary effect of APLF in breast cancer, which further lead to the decrease in tumor growth.

Recent reports suggest that induced expression of DNA repair factor associate with cancer metastasis [6]. Expressions of a panel of 34 DNA-repair genes from two different studies on breast primary tumor were significantly increased in tumors of metastatic origin [6]. As APLF knockdown could impede the metastatic behavior of MDAMB-231 cells (Fig. 2j-l), we determined the expression of these DNA repair related genes in response to APLF downregulation. GSEA of these genes clustered them into different repair pathways including Mismatch Repair (MMR), Base Excision Repair (BER), Homologous Recombination (HR), DNA replication, Nucleotide Excision Repair and p53-signaling pathway (Additional file 2) [6]. Representative genes from all pathways were screened and upon APLF downregulation, 7 genes including *BRCA1*, *FANCG, PCNA*, *MSH5*, *TERF1*, *RAD21* and *UBE2V2* were significantly downregulated whereas *CRY2* and *SMC4* were upregulated while other repair genes remained unaltered (Fig. 2m). Thus, APLF mediated regulation of DNA repair genes could further contribute to breast cancer metastasis.

### APLF regulate genes in EMT

**Fig. 3:**
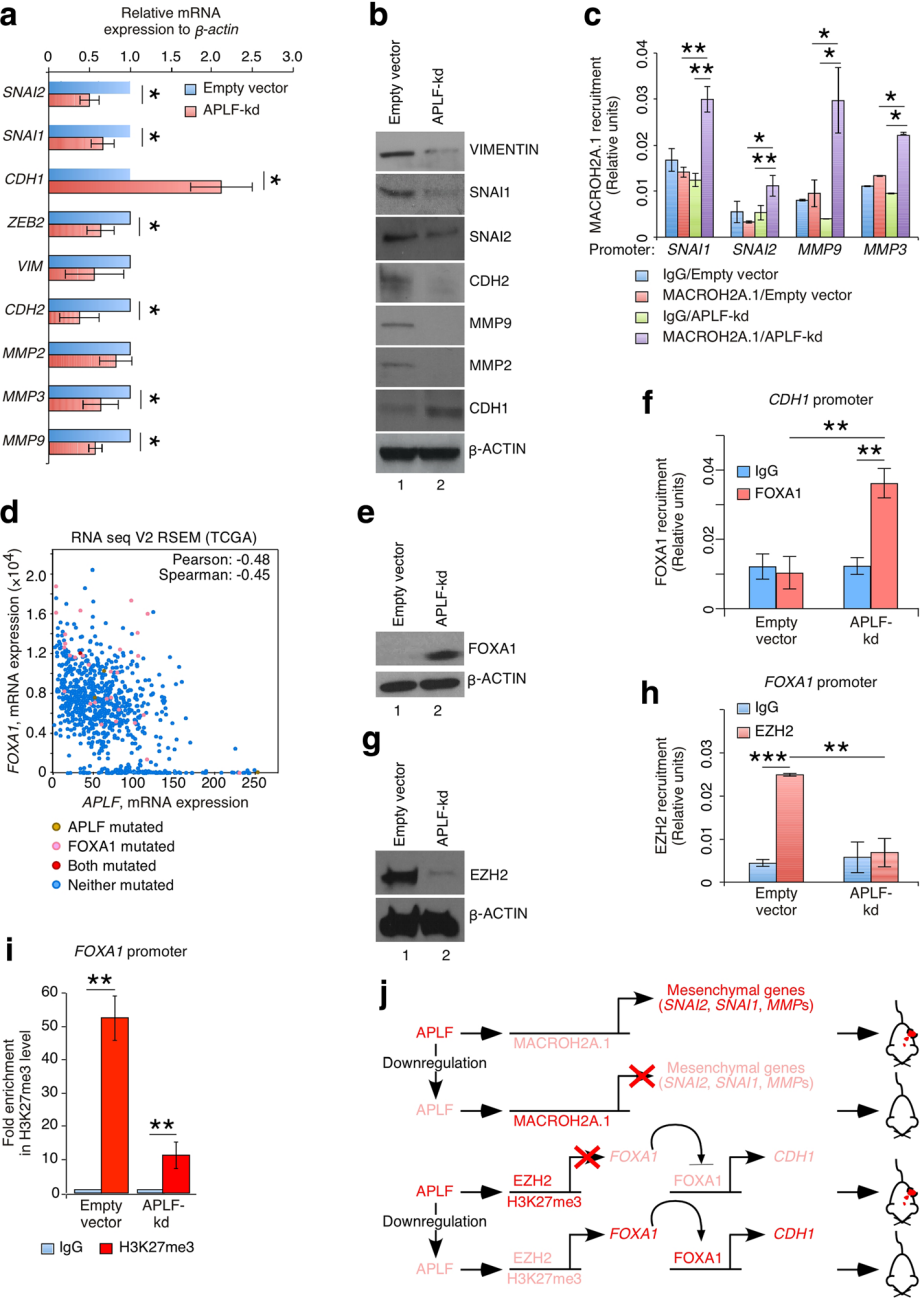
APLF regulate EMT. **a**, **b** Control and *APLF*-kd MDAMB-231 cells were investigated for the expression of different genes implicated in EMT. mRNA and protein were extracted and analyzed for the expression of genes by qRT-PCR and western blot respectively. **c** Chromatin Immunoprecipitation (ChIP) analysis was performed with control and *APLF*-kd MDAMB-231 cells for the recruitment of MACROH2A.1 at EMT-specific gene promoters. Enrichment of chromatin fragments was measured by qRT-PCR using Sybr green fluorescence relative to a standard curve of input chromatin. IgG was used as the negative control [1]. **d** Co-expression analysis at the mRNA level between *APLF* and *FOXA1* in samples from TCGA study [5]. Co-expression analysis demonstrated maximal negative correlation of *APLF* with *FOXA1* expression supported by a Pearson score of − 0.48. **e** Protein was extracted from control and *APLF*-kd MDAMB-231 cells and analyzed for the expression of FOXA1 by western blot. **f** ChIP analysis was performed with control and *APLF*-kd MDAMB-231 cells. The plots represent the recruitment of FOXA1 at *CDH1* promoter. IgG was used as the negative control. Enrichment of chromatin fragments was measured by qRT-PCR using Sybr green fluorescence relative to a standard curve of input chromatin. **g** Expression of EZH2 in control and *APLF*-kd MDAMB-231 cells at protein level was analyzed by western blot. **h** ChIP analysis was performed with control and *APLF*-kd MDAMB-231 cells. The plots represent the recruitment of EZH2 at endogenous *FOXA1* promoter. IgG was used as the negative control. I. Same set of cells analyzed in H were investigated for the incorporation of H3K27me3 mark at endogenous *FOXA1* promoter. The graph represents the fold enrichment with respect to the input. IgG was used as the negative control. **j**. Model depicting the mechanism responsible for downregulation of mesenchymal genes and upregulation of epithelial gene *CDH1* in response to APLF downregulation in TNBC MDAMB-231 cells. Error bar = S.E.M for three independent experiments. Statistical analyses were performed using Student t-Test function, *p < 0.05, **p < 0.01

In order to metastasize, epithelial cells dislodge into blood circulation from primary tumors by invading through the basal lamina. EMT has been attributed to these phenomena and is often guided by a set of genes. Upon APLF-knockdown, EMT-specific markers including SNAI1, SNAI2 were downregulated whereas MET-favoring CDH1 level was significantly upregulated (Fig. 3a, b). An EMT gene set analysis from Molecular Signature database (Gene ontology, GO:0001837) [7] for TCGA invasive breast carcinoma study [5] showed majority of EMT-specific genes correlated to the upregulation of *APLF* (Additional file 1: Figure S7A). Individual assessment of genes, not included in the original study [7] (Additional file 3) demonstrated significant correlation in expression of EMT-genes with *APLF* (Additional file 1: Figure S7B). Ectopic expression of *APLF* in MDAMB-231 cells resulted in enhanced expression of EMT-specific genes (Additional file 1: Figure S8).

But, mechanistically how APLF could regulate these genes? On this context, we explored the role of the histone variant MacroH2A.1 associated with APLF during reprogramming and DNA damage induced repair [1, 2]. Histone variant MacroH2A.1 represent compact chromatin resulting in repressed locus. We observed that *MacroH2A.1*, encoded by *H2AFY*, expression increased upon downregulation of APLF both at the mRNA and protein level, but statistically insignificant (Additional file 1: Figure S9A, S9B), although, TCGA study [5] analysis showed significant downregulation of *H2AFY* in patients with increased *APLF* expression (Additional file 1: Figure S7B). So, we determined MACROH2A.1 recruitment at promoter of EMT-specific genes. We observed that *SNAI1, SNAI2, MMP3* and *MMP9* promoters were significantly enriched with MACROH2A.1 level in *APLF*-kd cells as compared to control MDAMB-231 cells (Fig. 3c). The repressed loci of EMT-related genes further conform to our observation in downregulation of these genes in *APLF*-kd MDAMB-231 cells (Fig. 3a, b). But, no significant loss in MACROH2A.1 was observed at the *CDH1* promoter in *APLF*-kd cells (Additional file 1: Figure S9C), which indicated the presence of an additional mechanism operating in regulation of CDH1 as a function of APLF level.

Forkhead box protein A1 (FOXA1) belongs to the group of forkhead transcription factors and termed as “pioneer factor” in breast cancer [8]. FOXA1 repression shifts breast cancer subtype from luminal to basal while its expression restricts metastasis of luminal subtype by inducing CDH1 level [9]. Interestingly co-expression analysis of invasive breast carcinoma samples in TCGA [5], revealed highest negative Pearson score of *APLF* with *FOXA1* (Pearson score = − 0.48, Fig. 3d). FOXA1 expression was upregulated in *APLF*-kd cells (Fig. 3e). It is known that FOXA1 influence chromatin reorganization by binding to heterochromatin and thereby facilitating expression of the gene relieved from compaction [8]. Expectedly, we failed to observe FOXA1 recruitment at the conserved *CDH1* promoter [10] in control MDAMB-231 cells whereas a significant enrichment of FOXA1 was detected in *APLF*-kd cells (Fig. 3f). So downregulation of APLF enhanced CDH1 expression by recruitment of induced FOXA1 at *CDH1* promoter and hence might restrict EMT of MDAMB-231 cells.

But, how APLF could regulate FOXA1? No significant difference in MACROH2A.1 recruitment at *FOXA1* promoter was observed in control and *APLF*-kd cells (Additional file 1: Figure S9D). Then how binding of FOXA1 could be facilitated under this condition? We reasoned that FOXA1 being “pioneer factor” could bind to its target site even in compact state of the chromatin [8]. Additionally, FOXA1 recruitment is facilitated by different histone modification marks including histone H3K4me2 [8]. Interestingly, during reprogramming of MEFs to iPSCs, APLF-downregulation induced H3K4me2 level [1]. This might be an additional contribution towards an enhanced recruitment of FOXA1 at the *CDH1* promoter in *APLF*-kd MDAMB-231 cells. But, eventually, what drives the upregulation of FOXA1 in *APLF*-kd cells? Loss in FOXA1 expression in metastatic MDAMB-231 cells corresponded to its hypermethylated promoter including both repressive histone H3K27me3 level and DNA methylation [9]. Histone chaperones in nature could interact with histone modifying enzymes and thereby could modulate different histone modification patterns. Histone H3K27 is tri-methylated by components of Polycomb Repressor Complex2, namely EZH2 and EZH1. Among them, Enhancer of Zeste2 (EZH2), has been implicated in metastasis and invasiveness of breast cancer. So, we studied the EZH2/H3K27me3 axis in regulation of FOXA1 as a function of APLF. TCGA sample analysis for invasive breast cancer [5], demonstrated a positive correlation of *EZH2* expression in patients with upregulated *APLF* expression (Additional file 1: Figure S7B). Upon downregulation of APLF, EZH2 expression was reduced both at the mRNA and protein level in MDAMB-231 cells (Additional file 1: Figure S9E) (Fig. 3g). We observed that APLF-knockdown could significantly enhance the recruitment of MACROH2A.1 at the *EZH2* promoter in MDAMB-231 cells (Additional file 1: Figure S9F). This could account for the loss in EZH2 expression. To investigate further, we studied the recruitment of EZH2 at the endogenous *FOXA1* promoter [9]. *FOXA1* promoter in control MDAMB-231 cells was significantly enriched with EZH2 whereas no recruitment was observed in APLF-kd cells (Fig. 3h) (Additional file 1: Figure S9G). No change in the global level of H3K27me3 was observed in control and *APLF*-kd MDAMB-231 cells (Additional file 1: Figure S9H). It should be noted here that EZH2 and EZH1 functions are complementary and sometimes redundant. RNA-seq analysis of TCGA samples [5] demonstrated positive correlation of *APLF* with *EZH2* while negatively correlated with *EZH1* (Additional file 1: Figure S7B) So upon downregulation of APLF in MDAMB-231 cells, contrasting EZH2 and EZH1 level might have resulted in an unaltered global H3K27me3 level. But, APLF-downregulation significantly reduced the fold enrichment of H3K27me3 mark at the *FOXA1* promoter in comparison to control MDAMB-231 cells (Fig. 3i). Loss in H3K27me3 renders an open chromatin resulting in increased expression of the gene. Thus, absence of EZH2 recruitment followed by loss in repressive H3K27me3 level, upon downregulation of APLF resulted in enhanced expression of FOXA1.

## Conclusion

Here, we provided novel evidence for enrichment of APLF in breast tumors, which could regulate metastasis-associated EMT in invasive breast cancer (Fig. 3j).

## Additional files

Additional file 1: Material & methods, Supplementary Figures, Tables. (ZIP 4889 kb)

Additional file 2: GSEA of genes induced in breast cancer metastasis among different repair pathways related to Fig. 2M. (XLSX 42 kb)

Additional file 3: Expression of EMT associated genes related to Figure S7B. (XLSX 10 kb)

## Abbreviations

ANT: Adjacent normal tissue
APLF: Aprataxin PNK-like Factor
BRCA1: Breast Cancer 1
CDH1: Cadherin 1
CRY2: Cryptochrome-2
EMT: Epithelial-to-mesenchymal transition
EXO1: Exonuclease 1
EZH1: Enhancer Of Zeste 1
EZH2: Enhancer of zeste homolog 2
FANCG: Fanconi anemia group G
FOXA1: Forkhead Box A1
GSEA: Gene Set Enrichment Analysis
IDC: Invasive ductal carcinoma
IHC: Immunohistochemistry
kd: knockdown
MAD2L1: Mitotic Arrest Deficient 2 Like 1
MSH5: MutS Homolog 5 (*E. Coli*)
NHEJ: Non-homologous End Joining
NOD/SCID: Non-obese diabetic/Severe Combined Immunodeficiency
PCNA: Proliferating cell nuclear antigen
PNK: Polynucleotide Kinase
POL8: DNA Polymerase Beta
RAD21: Double-strand-break repair protein rad21 homolog
RCF4: Replication Factor C Subunit 4
RRM2: Ribonucleotide Reductase Regulatory Subunit M2
SHFM1: Split Hand/Foot Malformation Type 1
SMC2: Structural Maintenance Of Chromosomes 2
SMC4: Structural Maintenance Of Chromosomes 4
SNAI1/2: Snail Family Transcriptional Repressor 1/2
TCGA: The Cancer Genome Atlas
TERF1: Telomeric Repeat Binding Factor 1
TNBC: Triple Negative Breast Cancer
UBE2V2: Ubiquitin-Conjugating Enzyme E2 Variant 2
