# Supplementary material for: Enhanced expression of histone chaperone APLF associate with breast cancer
Source: Mol Cancer. 2018 Mar 26;17:76. doi: 10.1186/s12943-018-0826-9 (PMC5870250; doi:10.1186/s12943-018-0826-9)
Supplement: Supplementary file 1 — Material & methods, Supplementary Figures, Tables. (ZIP 4889 kb) [file 12943_2018_826_MOESM1_ESM.zip › Additional file 1.docx]

**Enhanced expression of Histone chaperone APLF associate with breast cancer**

**Supplementary Information**

**Materials & methods**

**Figure legends**

**Tables**

**Materials & methods**

**Cell culture**

HEK 293T, MDAMB-231, MCF7, SUM149, SKBR3, T47D, SW480, MDAMB-468 and SW620 cells were grown in DMEM (Invitrogen; 12440053) containing 10% FBS (Invitrogen; 1600044) and 1% Penicillin/Streptomycin (Invitrogen; 10378016), 1% Antimycotic/Antibiotic (Invitrogen; 15240062). MCF10A cells were grown in MEBM with additives (MEGM^TM^ Mammary Epithelial Cell Growth Medium Bullet kit^TM^; Lonza; CC3150).

**Animal studies**

Animal-related experiments were performed at the host institute, according to the Committee for the Purpose of Control and Supervision of Experiments on Animals (CPCSEA) guidelines and institutional protocol approved by Institutional Animal Ethics Committee (#IAEC/265/DSD/2014). NOD SCID mice (6–8-week-old females) (n=3) were used for the generation of tumor.

**Immunofluorescence**

Immunostaining to detect the expression of APLF and γH2AX were performed using standard protocols [1]. Cells were incubated with primary antibodies overnight and fluorescent conjugated secondary antibodies (Alexa Fluor 488; Invitrogen; A11008) were used. Cells were co-stained with Hoechst dye (Sigma; B1155, 1μg/ml) for nuclear staining. Images were captured by confocal microscopy.

**Western Blot analysis**

Cell pellets were lysed in RIPA buffer [2] and Bradford reagent (Bio- Rad; 500-0006) was used to determine the protein concentrations and samples were separated by SDS-PAGE. List of antibodies have been enlisted in Additional file 1, Table S1.

**Quantitative real time PCR (qRT-PCR) analysis**

Total RNA was isolated using Qiagen RNeasy Kit (Qiagen; 74106) according to manufacturer’s protocol. cDNA was prepared by a high capacity cDNA reverse transcription kit (ABI; 4368814). Power Sybr green master mix (ABI; 4367659) was used for quantitative RT-PCR analysis. Primer sequences have been enlisted in Additional file 1, Table S2.

**RNA Interference and generation of stable APLF-knocked down cells**

Human *APLF* shRNA (5’ CATCCTGGTGATAGTGATTAT 3’) used by Ahel’s lab [3] was used to target the gene. Lentiviral vectors containing the shRNA was cloned in the Plko.1 (Addgene) vector. Lentiviral supernatant was produced in HEK 293T cells by transient transfection using calcium chloride following the protocol described earlier [1, 2]. MDAMB-231 cells were transfected with viral particles and screened for the generation of stable cells in presence of puromycin (Sigma; P8833). After 3 days of selection, RNA and protein were extracted for analysis. Quantitative RT-PCR and western blotting confirmed the knockdown of APLF.

**Wound Closure assay**

Cancer cells were plated in a 6-well plate coated with 0.1% gelatin and cultured overnight in serum starved condition (2% FBS). Next day, 10μg/ml of mitomycin C (Sigma; M4287) was added to the culture to inhibit proliferation of the cells and incubated for 2h. Scraping the cell monolayer with a 20μl pipette tip created wound. Medium and non-adherent cells were removed, and cells were washed twice with PBS, and fresh medium was added. Cells were permitted to migrate into the area of clearing for different time points. Wound closure or wound healing was monitored by visual examination under the microscope. Percentage wound recovery was expressed in terms of [1–(Width of the wound at a given time/width of the wound at t=0)] × 100% [4].

**Matrigel invasion assay**

Invasion assay was performed in invasion chamber from Corning (Corning® BioCoat™ Matrigel® Invasion Chamber; 354480). In 24-well invasion chamber, 2x10^4^ control, *APLF*-kd MDAMB-231 cells and other cancer cells were introduced into the upper chamber while 10% FBS was placed in the lower chamber. The cells were allowed to invade the membrane for 24h. Percentage of invaded cells was calculated by Hoechst staining for the nucleus to the total number of cells added to the upper chamber.

**Cytotoxicity/Viability assay**

5000 MDAMB-231 (control and *APLF-*kd) cells were plated in 96 well plate for overnight incubation. Next day, 20 μl of 5mg/ml MTT (Sigma; M2128) in 100μl media was added in cells and incubated for 1h in 37ºC incubator in dark. Supernatant was removed and 100μl DMSO (Sigma; D2650) was added and incubated for 30 minutes in shaking condition at room temperature. Absorbance was measured at 490nm with reference 600 nm.

**Cell cycle analysis**

Control and APLF-kd cells were synchronized at the G0/G1 phase by serum starvation [5]. 1×10^6^ MDAMB-231 cells (control and *APLF*-kd) were washed with PBS and fixed in 70% ethanol. Then cells were treated with RNase A (Sigma; P4170) and incubated with propidium iodide (PI) (Sigma; P4170), followed by analysis on BD FACS AviaTM II instrument.

**DNA damage and recovery assay**

Control and *APLF*-kd MDAMB-231 cells were treated with 10μM etoposide (Sigma; E1383) for 4h. Etoposide was removed and cells were thoroughly washed with 1X PBS and allowed to recover in culture medium for different time points. Recovered cells were analyzed for the presence of γH2AX (Abcam; ab2893) foci by IF study.

**Tumor generation in NOD-SCID mice**

Control and *APLF*-kd MDAMB-231 cells were subcutaneously injected (10^6^ cells in 100 μl 1X PBS) in NOD SCID mice. Tumor dimensions were measured twice per week using a vernier caliper. Primary tumors with adherent tissue were excised, fixed in 10% formalin/PBS, and embedded in paraffin. Tissue sections (5 μm) were prepared and stained with H&E. Tumor sections were examined to assess tumor invasiveness.

**Immunohistochemistry**

Tissues were dissected, fixed in 10% buffered formalin (Sigma; HT501128), and embedded in paraffin. Paraffin sections were deparaffinized in xylene (Sigma; 534056) and rehydrated through a series of alcohol wash. For antigen retrieval, sections were boiled for 20 min in citrate buffer pH-6 (Santacruz; sc294091) and cool down to room temperature. Sections were washed with distilled water and draw the section area with a hydrophobic pen (IHC World; SPR0905). After blocking with endogenous blocking solution [3%H2O2 (Fischer Scientific; H325100) in methanol], washed with 1X PBS and further blocked with protein blocking solution provided by kit (Dako RealTM Envision; 15007). Sections were incubated with primary antibody (APLF #1 and #2-1:100) overnight at 4ºC. HRP-conjugated secondary antibody raised against rabbit IgGs were used. After PBS wash, sections were incubated with DAB for 3 min until color developed. Hematoxylin (Sigma; H3136) counterstaining was performed until color developed and washed with water. Slides were finally washed with 1X PBS and the mounted with DPX (Merck).

**Ectopic expression of *APLF***

Human *Aplf* cDNA (Accession No: BC041144; cDNA clone MGC #47799) purchased from the transOmics technologies was PCR amplified and subcloned into pEGFP-C1 (Clonetech, Catalog #6084-1) at *BamH*1 and *EcoR*1 sites.

***In vivo* metastasis assay**

~10^6^ control and APLF-kd MDAMB-231 cells were injected into the lateral tail vein of NOD-SCID mice (n=3 for each group; age= 6-8 weeks). Prior to this, control and *APLF*-kd MDAMB were transfected with pEGFPC1 (Clonetech; 6084-1). by lipofectamine 2000 (Invitrogen; 11668019). Cells were selected in presence of 500μg/ml of G418 (Sigma; G8168) for 3 weeks, sorted by FACS and propagated further. Regular monitoring of cell invasion was detected by IVIS^R^ Spectrum (Parkin Elmer). After 6 weeks of injection, lungs were dissected and examined for the presence of metastatic nodules. The lungs were preserved for further analyses.

**RNA isolation from tissue sample**

50mg of lung samples (mentioned above) were homogenized in sterile homogenizer, followed by the addition of 1ml TRIzol (Invitrogen; 15596026). After homogenization samples were taken in a fresh microfuge tube and kept at -80°C for 1 hour. Next the samples were thawed on ice. 200 μl chloroform was added and vortexed for 20 seconds. The samples were incubated on ice for 3minutes and centrifuged at 12,000g for 15 min at 4°C.The aqueous layer was precipitated with equal volume of isopropanol, incubated at room temperature for 1 hour and centrifuged at 12,000g for 15 minutes at 4°C.The pellet was washed with 75% ethanol and centrifuged at 10,000 rpm for 10 minutes at 4°C. Supernatant was discarded and the pellet was resuspended in 20μl nuclease free water.

**Generation of shRNA resistant MDAMB-231 cells**

Mutated variants of the *APLF* shRNA (mentioned above) was generated by site directed mutagenesis using the primer 5′-attttagccatcctggagactccgactatggaggtgtac-3′ to introduce six silent mutations (underlined) in the coding region targeted by the shRNA, as mentioned above. Site-directed mutagenesis was approached by megaprimer synthesis using protocol described earlier [6, 7]. Mutated *APLF* cDNA was cloned into pEGFPC1 at *EcoR*I and *BamH*I sites and was transfected in MDAMB-231 APLF-kd cells by Lipofectamine 2000 (Invitrogen; 11668019). After three days, cells were analyzed for the expression of APLF and survivability by MTT assay.

**Chromatin Immunoprecipitation (ChIP)**

Briefly, 10^6^ cells were trypsinized, crosslinked with formaldehyde (1%), and sonicated to generate chromatin fragments. Antibodies were used to immunoprecipitate protein-DNA cross-linked fragments. Precipitated complexes were eluted and reverse crosslinked. Enrichment of chromatin fragments was measured by qRT-PCR using Sybr green fluorescence relative to a standard curve of input chromatin. IgG was used as the negative control [1]. List of primers have been enlisted in Additional file 1, Table S3.

**Apoptosis assay**

Cells were stained with Annexin-V- FITC and propidium iodide according to the manufacturer protocol (Sigma; APOAF-20TST), followed by flow cytometry analysis by BD FACS AviaTM II instrument.

**Reference**:

1. Majumder A, Syed KM, Joseph S, et al. Histone Chaperone HIRA in Regulation of Transcription Factor RUNX1. J Biol Chem*.* 2015;290:13053-63.

2. Syed KM, Joseph S, Mukherjee A et al. Histone chaperone APLF regulates induction of pluripotency in murine fibroblasts. J Cell Sci. 2016;129:4576-4591.

3**.** Mehrotra PV, Ahel D, Ryan DP, et al. DNA repair factor APLF is a histone chaperone. *Mol Cell*. 2011;41:46-55.

4. Chen J, Miller EM, Gallo KA. MLK3 is critical for breast cancer cell migration and promotes a malignant phenotype in mammary epithelial cells. Oncogene. 2010;29: 4399-4411.

5. Yang X, Sun Y, Li H, et al. C-terminal binding protein-2 promotes cell proliferation and migration in breast cancer via suppression of p16INK4A. Oncotarget. 2017;8:26154-26168.

6. Sambrook J, Russell DW. *CSH Proto*c. 2006;2006(1).

7. Tyagi R, Lai R, Duggleby RG. *BMC Biotechnol*. 2004;4:2.

8. Timmerman LA, Grego-Bessa J, Raya A, Bertrán E, Pérez-Pomares JM, Díez J, et al. Notch promotes epithelial-mesenchymal transition during cardiac development and oncogenic transformation. Genes Dev. 2004;18:99-115.

**Supplementary Figure legends:**

**Figure S1.** A. IHC analysis for the expression of APLF in invasive breast tumor section using APLF antibody from commercial source (upper panel, APLF#1) and gift from Prof. Ivan Ahel [3] (lower panel, APLF#2). Invasive ductal breast tumor sections and normal adjacent tissue sections (n=2) were procured from Regional Cancer Centre (RCC), Thiruvananthapuram, Kerala, India. The protocol received human ethical clearance from RCC (#HEC 30/2006). Representative section from one of the patient has been included in the figure. B. Immunofluorescence study for the expression of APLF in the same tissue sections described above (APLF antibody #1 was used).

**Figure S2. APLF expression associate with invasive nature of breast cancer cells.** A. MCF10A, MCF7 and MDAMB-231 were used as normal, non-metastatic and metastatic cell line respectively. mRNA and protein expression was analyzed by quantitative RT-PCR and western blot. Error bar= S.E.M for three independent experiments. Statistical analysis was performed using Student t-Test function, *p<0.05, **p<0.01. Band intensity was measured by ImageJ software (RBI=Relative Band Intensity). A representative image for the blot has been presented. B. Migratory potential for three different breast cancer cell lines with distinct difference in APLF expression were analyzed. Bar graph represents percentage of wound recovery expressed in terms of [1-(Width of the wound at a given time/width of the wound at t=0)]^2^ for three different cell lines**.** C. The same set of cells analyzed in B were subjected to invasion assay to determine their invasive potential in comparison to the level of APLF expression. The graph represent the percentage of cells invaded and expressed in terms of number of cells invaded to total number of cells added to the upper chamber at the start of the experiment. D. APLF expression was re-confirmed in different breast cancer cell lines using APLF antibody #2 [3]. E. Analysis of microarray data from Cancer Cell Line Encyclopedia for *APLF* mRNA expression profile across the cancer cell lines originating from six primary adenocarcinomas that are commonly associated with invasion and metastasis. F. Protein was extracted from colon cancer cell lines and measured by Bradford reagent. Expression of APLF was determined by western blot analysis. Antibody enlisted in Supplementary Table 1.

**Figure S3.** A. Screenshot showing SpliceCenter, web-based online bioinformatics tool, analysis for the detection of off-targets of *APLF* shRNA. B. *APLF* full-length cDNA (Gene ID: 200558) was PCR amplified and subcloned into FLAG-HA-pcDNA3.1 vector (Addgene; 52535) and transfected into HEK293 cells. Control and APLF-FLAG transfected cells were analyzed for the expression of APLF by IF using standard protocol. C. Western blot analysis further confirmed the overexpression of APLF in HEK293 transfected with FLAG tagged-APLF.

**Figure S4. *APLF* overexpression influence cell cycle.** A.MCF10A cells were transfected with lentiviral particles expressiong APLF-shRNA or empty Plko.1 vector as described in the materials and methods section. Quantitative RT-PCR analysis demonstrated the knockdown of APLF in MCF10A. B. Control and APLF-kd MCF10A cells were subjected to cell cycle analysis. Bar graph represents the fraction of cells in each phases of the cell cycle. C. Phase contrast images of control and APLF-kd MCF10A cells demonstrated no phenotypic change. D. Construct used for cloning human *APLF* cDNA. Human *APLF* full length cDNA (Gene ID: 200558) was amplified by PCR. The amplified *APLF* cDNA was cloned into *Xba*I and *BamH*I sites in pEGFPC1 (Clonetech). E. MDAMB-231 cells were transfected with either pEGFPC1 (EGFPC1) or *APLF*-cDNA cloned into pEGFPC1 (EGFPC1/*APLF*). GFP positive cells were sorted by FACS and propagated for further analysis. qRT-PCR analysis demonstrated the over-expression of *APLF* in EGFPC1/*APLF* transfected cells. F. EGFPC1 and EGFPC1/*APLF* MDAMB-231 cell were treated with PI and subsequently analysed for the cell population present in different phases of cell cycle by FACS. Bar graph represents the percentage of cells in the different phases of the cell cycle in control and *APLF*-overexpressing MDAMB-231 cells. G. Quantitative ChIP was performed with control and *APLF*-kd cells to determine the recruitment of MACROH2A.1 at the *CYCLIN D1* promoter. IgG was used as the negative control. H. Apoptosis assay. Control (left panel) and APLF-kd (right panel) cells were stained with Annexin-V–FITC and propidium iodide, followed by FACS analysis. Q1 represents FITC-labeled apoptotic cells. Error bar=S.E.M for three independent experiments. Statistical analyses were performed using Student t-Test function, **p<0.01.

**Figure S5.** **Validation of *APLF* shRNA by** **derivation of *APLF* shRNA resistant MDAMB-231 cells**. Mutated variants of the shRNA [3] was generated by site directed mutagenesis to introduce six silent mutations in the coding region targeted by shRNA [3]. Site-directed mutagenesis was approached by megaprimer synthesis using protocol described earlier. Mutated *APLF* cDNA was cloned into pEGFPC1 at *EcoR*I and *BamH*I sites and was transfected in MDAMB-231 APLF-kd cells by Lipofectamine 2000. After three days, cells were analyzed for the expression of APLF (A) and survivability by MTT assay (B). C. APLF-shRNA resistant mutant cells were subjected to cell cycle analysis. Bar graph represents the fraction of cells in each phases of the cell cycle.

**Figure S6. *APLF* overexpression increased invasive nature.** A. Control and APLF-kd MDAMB-231 cells were subjected to apoptosis assay after 40h of culture. Bar graph represents the fraction of apoptotic cells. B. MDAMB-231 cells transfected with EGFPC1 or EGFPC1/*APLF* (mentioned in Additional File 1, Figure S4D, Figure S4E) were subjected to matrigel invasion assay. Percentage of cells invaded indicates induced invasive nature of EGFPC1/*APLF* cells. Error bar= S.E.M for three independent experiments. Statistical analyses were performed using Student t-Test function, *p<0.05.

**Figure S7.** A, B. Volcano plot represents the expression of EMT genes (GO:0001837) [8] with respect to *APLF* alteration at the mRNA level in the invasive breast carcinoma TCGA samples and other genes relevant to the study but not included in the original study (GO:0001837).

**Figure S8. APLF overexpression enhanced the expression of EMT-specific genes**. EGFPC1 and EGFPC1/*APLF* overexpressing MDAMB-231 cells were investigated for the expression of genes responsible for invasive and metastatic behavior or involved in EMT. qRT-PCR analysis demonstrated the enhanced expression of these genes. Error bar= S.E.M for three independent experiments. Statistical analyses were performed using Student t-Test function, **p<0.01. Primers enlisted in Additional file 1,Table S2.

**Figure S9**. A. Expression of MACROH2A.1 encoded by H2AFY in control and *APLF*-kd MDAMB-231 cells by qRT-PCR analysis. B. Same set of samples were analyzed for the expression of MACROH2A.1 by western blot. Band intensity was measured by ImageJ software. C, D. ChIP was performed with control and *APLF*-kd cells to determine the recruitment of MACROH2A.1 at the *CDH1* and *FOXA1* promoter respectively. IgG was used as the negative control. E**.** Expression of *EZH2* in control and *APLF*-kd MDAMB-231 cells at the mRNA level was analyzed by qRT-PCR. F. ChIP was performed with control and *APLF*-kd cells to determine the recruitment of MACROH2A.1 at the *EZH2* promoter. IgG was used as the negative control. G. Non-specific binding of the regulatory factors were analyzed at the *GAPDH* promoter. H. Control and *APLF*-kd MDAMB-231 cells were analyzed for the presence of H3K27me3 mark by western blot. Error bar=S.E.M for three independent experiments. Statistical analyses were performed using Student t-Test function, *p<0.05, **p<0.01. ChIP based primers enlisted in Table S3.

**Supplementary Tables**

**Table S1. Antibodies used in the study. Related to western blotting in the experimental procedures section.**

| Primary Antibody | Company | Catalog Number |
| --- | --- | --- |
| APLF (#1) | Sigma | SAB4500756 |
| N-Cadherin (CDH2) | Santacruz | sc 7939 |
| γH2AX | Abcam | ab2893 |
| APLF (#2) | Gift from Prof. Ivan Ahel Lab, Oxford University, UK | |
| FLAG | Abcam | ab1162 |
| VIMENTIN | Cell Signaling | 5741 |
| FOXA1 | Abcam | ab23738 |
| CYCLIN D1 | Santacruz | sc753 |
| MMP9 | Santacruz | sc21733 |
| MMP2 | Santacruz | sc13594 |
| MACROH2A.1 | Abcam | ab37264 |
| H3K27me3 | Abcam | ab6002 |
| EZH2 | Abcam | ab3748 |
| SNAI1 | Abcam | ab17732 |
| SNAI2 | Abcam | ab27568 |
| E-Cadherin (CDH1) | Abcam | ab76055 |
| Caspase 3 cleaved | Cell Signaling | 9661 |

| Secondary Antibody | Company | Catalog No |
| --- | --- | --- |
| Peroxidase Affinipure Goat Anti Rabbit IgG | Jackson Immuno Research | 111035144 |
| Peroxidase Affinipure Goat Anti Mouse IgG | Jackson Immuno Research | 115035062 |

**Table S2. Primers for qRT-PCR analysis (all sequences are human specific). Related to Quantitative RT-PCR in the experimental procedures section**

| Gene | Forward (5^’^-3^’^) | Reverse (5^’^-3^’^) |
| --- | --- | --- |
| *APLF* | CAGAAATAGCCAAGACCCAGA | GCTTATTGCAGTCTCTATTTTCACC |
| *ACTIN* | CCAGCTCACCATGGAGTATG | ATGCCGGAGCCGTTGTC |
| *CDH1* | TGCCCACAAAATGAAAAAGG | GTGTATGTGGCAATGCGTTC |
| *SNAI1* | AAGATGCACATCCGAAGCC | CGCAGGTTGGAGCGGTCAGC |
| *SNAI2* | ATACCACAACCAGAGATCCTCA | GACTCACTCGCCCCAAAGATG |
| *MMP3* | CTGGACTCCGACACTCTGGA | CAGGAAAGGTTCTGAAGTGACC |
| *TWIST1* | AAGCTGAGCAAGATTCAGACC | TGGAGGACCTGGTAGAGGAA |
| *ZEB2* | AACAACGAGATTCTACAAGCCTC | TCGCGTTCCTCCAGTTTTCTT |
| *CDH2* | CACTGCTCAGGACCCAGAT | TAAGCCGAGTGATGGTCC |
| *MMP9* | TGTACCGCTATGGTTACACTCG | GGCAGGGACAGTTGCTTCT |
| *VIM* | CAGGTGGACCAGCTAACCAA | CCTCTCTCTGAAGCATCTCCTC |
| *MMP2* | CCCACTGCGGTTTTCTCGAAT | CAAAGGGGTATCCATCGCCAT |
| *TERF1* | TGCTTTCAGTGGCTCTTCTG | ATGGAACCCAGCAACAAGAC |
| *RAD21* | AAGCCAAATACCTTCTTGCAGAC | CTGCTTCCCGATTTTCCTCAG |
| *RCF4* | CCGCTGACCAAGGATCGAG | AGGGAACGGGTTTGGCTTTC |
| *RRM2* | TTGAAACGASTGCCTTGTGTCA | ACCATAGGTAGCCTCTTTGTCC |
| *SHFM1* | GAAAAAGCAGCCGGTAGACTT | ATCCCAATTATCCTCCCAGACA |
| *SMC4* | CGCCTCCAGCAATGACCAAT | CCCCAGCATAGGATTTGAAGTT |
| *FANCG* | TAGGCTCTATCAGCAACTGGG | AAACTGCGGGGCTTTGGAA |
| *UBE2V2* | AGTTCCTCGTAATTTTCGCTTGT | CCCCAGCTAACTGTACCGT |
| *CRY2* | TCCCAAGGCTGTTCAAGGAAT | TGCATCCCGTTCTTTCCCAAA |
| *EXO1* | CCTCGTGGCTCCCTATGAAG | AGGAGATCCGAGTCCTCTGTAA |
| *MAD2L1* | GTTCTTCTCATTCGGCATCAACA | GAGTCCGTATTTCTGCACTCG |
| *POL8* | CCGCAGGAGACTCTCAACG | GTACTTGTGGATAGCTTGGCTC |
| *BRCA1* | TGTGAAGGCCCTTTCTTCTG | TCCCATCTGTCTGGAGTTGA |
| *PCNA* | CTCTTGCCTTACGCAAGTCTCA | GTCCTTGAGTGCCTCCAACA |
| *MSH5* | AGTGACTCCACTATCCACTTCA | ACTGGGGATTGATCTCATCCA |
| *SMC2* | ACAACACCAGAGTACAGGATCT | CGGCCCTGCATGATGAGAA |
| *H2AFY* | CTGTGGCCAATGATGAAGAG | AACTCGGGGTGGATGTTG |
| *EZH2* | ATGCGACTGAGACAGCTCAA | CGCTGTTTCCATTCTTGGTT |

**Table S3. Primers for q-ChIP analysis. Related to Chromatin Immunoprecipitation (ChIP analysis) in the experimental procedures section**

| Promoter | Forward (5^’^– 3^’^) | Reverse (5^’^ – 3^’^) |
| --- | --- | --- |
| *SNAI1* | AGTGGTTCTTCTGCGCTACT | GAAAGAGCGCGGCATAGTG |
| *SNAI2* | AGCATTTCAACGCCTCCAAA | TGTATGTGTGTCCAGTTCGC |
| *CDH1* | CTCTCAGTGGCGTCGGAA | GCGGGCTGGAGTCTGAAC |
| *CDH1* (FOXA1 binding site) [19] | CTCCAGCTTGGGTGAAAGAG | GGCCTTTTACACTTGGCTGA |
| *FOXA1 (*EZH2/H3K27me3 enrichment) [18] | AACAAGAAGATTTTGCGGATTC | GACCGGGTGATAGGCAATTA |
| *MMP3* | ACAGCAAGGCATAGAGACAACA | CACAGCAACAGTAGGATTGGA |
| *MMP9* | TAAAGCCCCCACAACAGCA | TCATGGTGAGGGCAGAGG |
| *FOXA1* | AACAAGAAGATTTTGCGGATTC | GACCGGGTGATAGGCAATTA |
| *GAPDH* | CTAGGCGCTCACTGTTCTCTC | CACCTTCCCCATGGTGTCT |
| *EZH2* | ATGGGCCAGACTGGGAAGA | CTTCCGCCAACAAACTGG |
| *CYCLIN D1* | TGTGCTGCGAAGTGGAAA | GGTCGTTGAGGAGGTTGG |
