# Supplementary figures and images for: Enhanced expression of histone chaperone APLF associate with breast cancer
Source: Mol Cancer. 2018 Mar 26;17:76. doi: 10.1186/s12943-018-0826-9 (PMC5870250; doi:10.1186/s12943-018-0826-9)

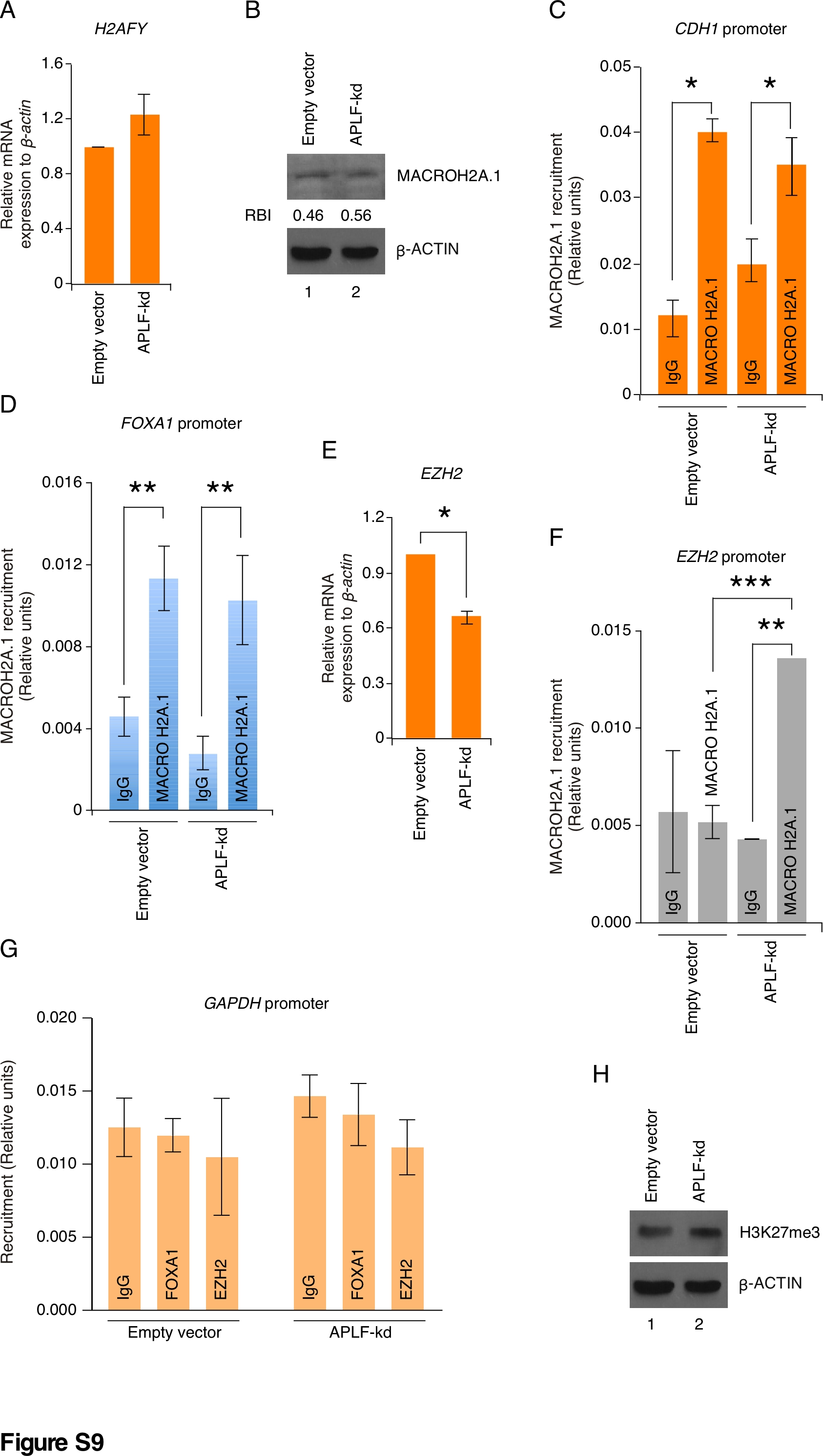

Supplement: Supplementary file 1 — Material & methods, Supplementary Figures, Tables. (ZIP 4889 kb) [file 12943_2018_826_MOESM1_ESM.zip › Figure S9.jpg]

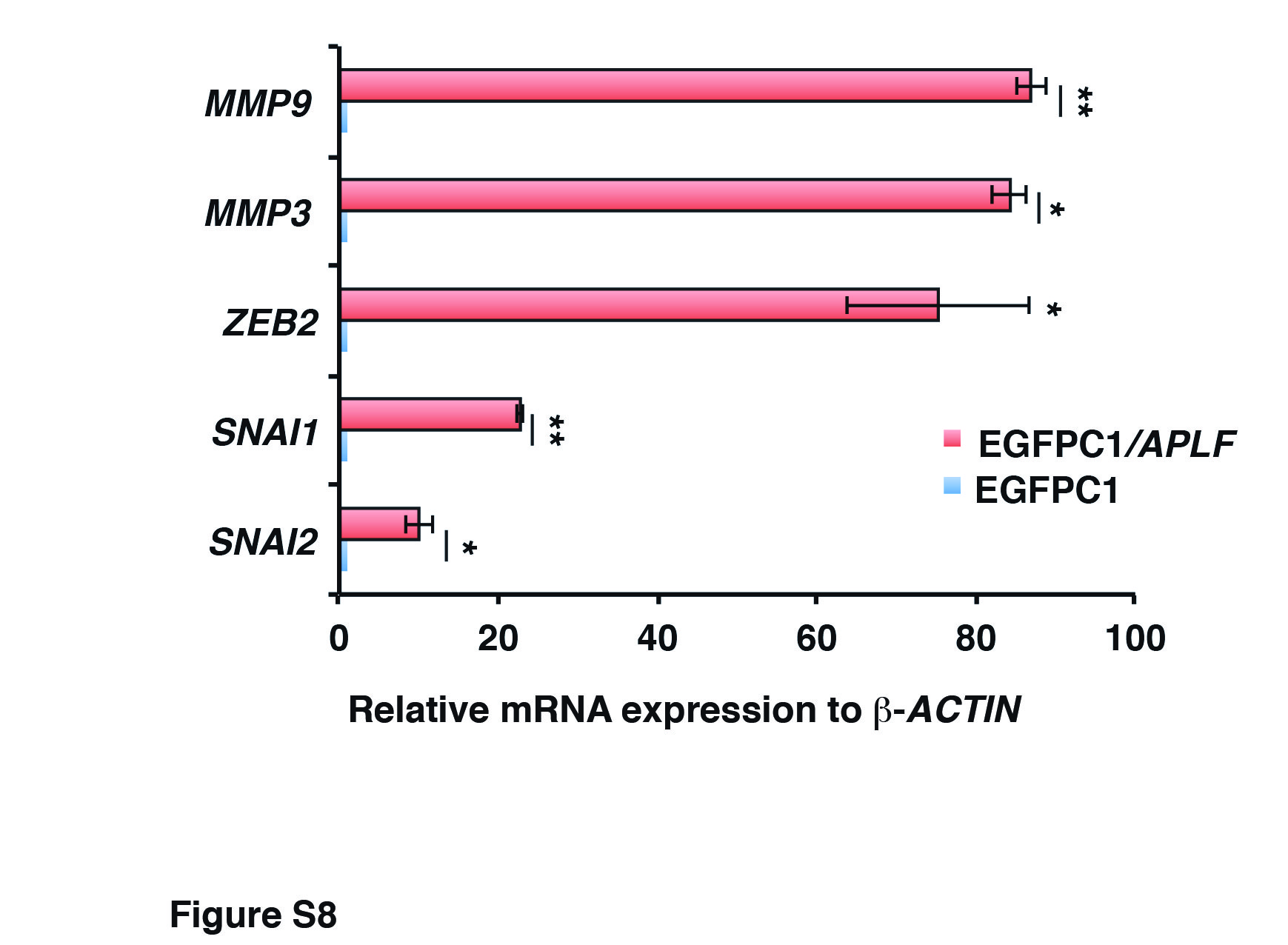

Supplement: Supplementary file 1 — Material & methods, Supplementary Figures, Tables. (ZIP 4889 kb) [file 12943_2018_826_MOESM1_ESM.zip › Figure S8.jpg]

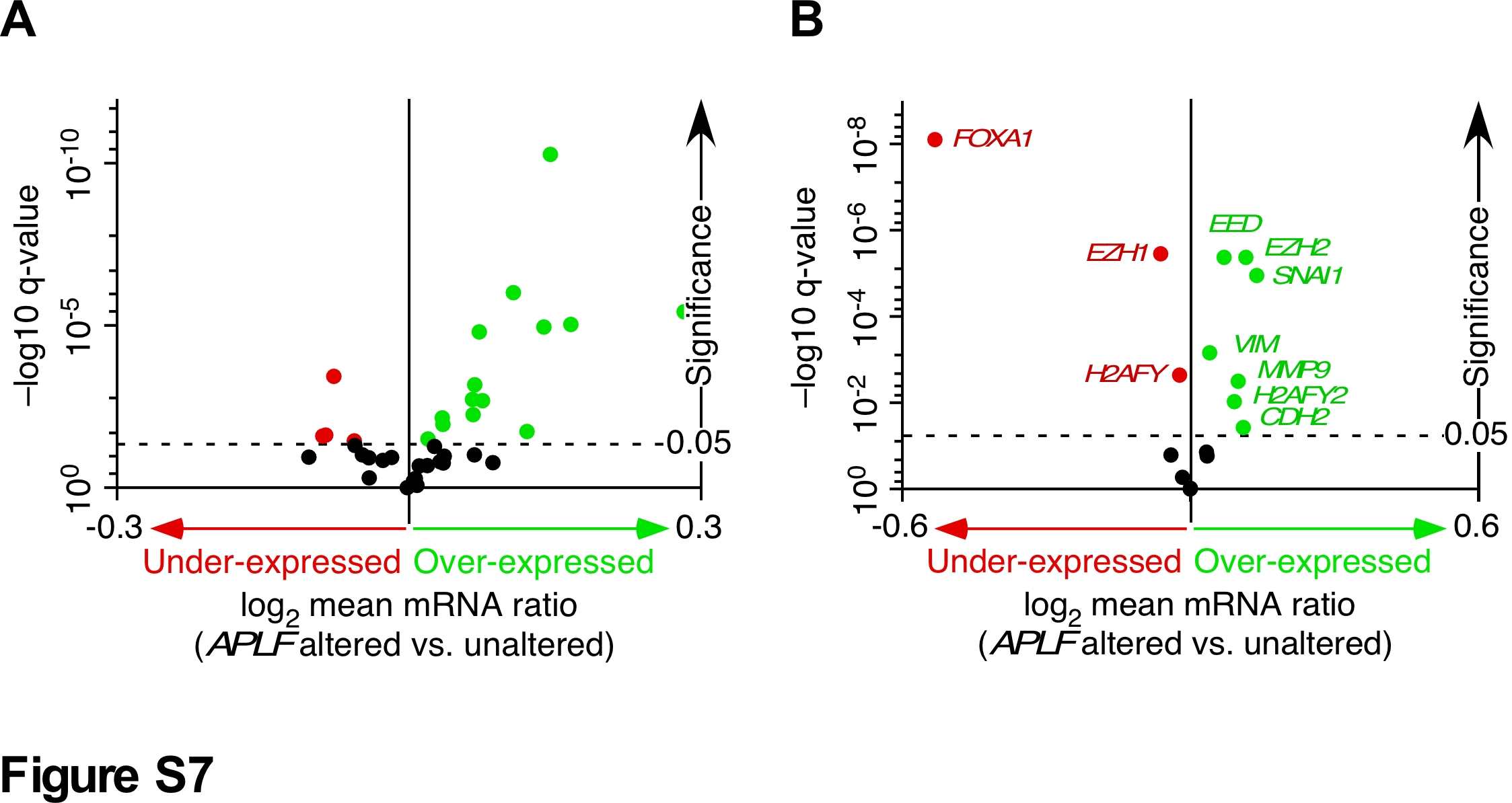

Supplement: Supplementary file 1 — Material & methods, Supplementary Figures, Tables. (ZIP 4889 kb) [file 12943_2018_826_MOESM1_ESM.zip › Figure S7.jpg]

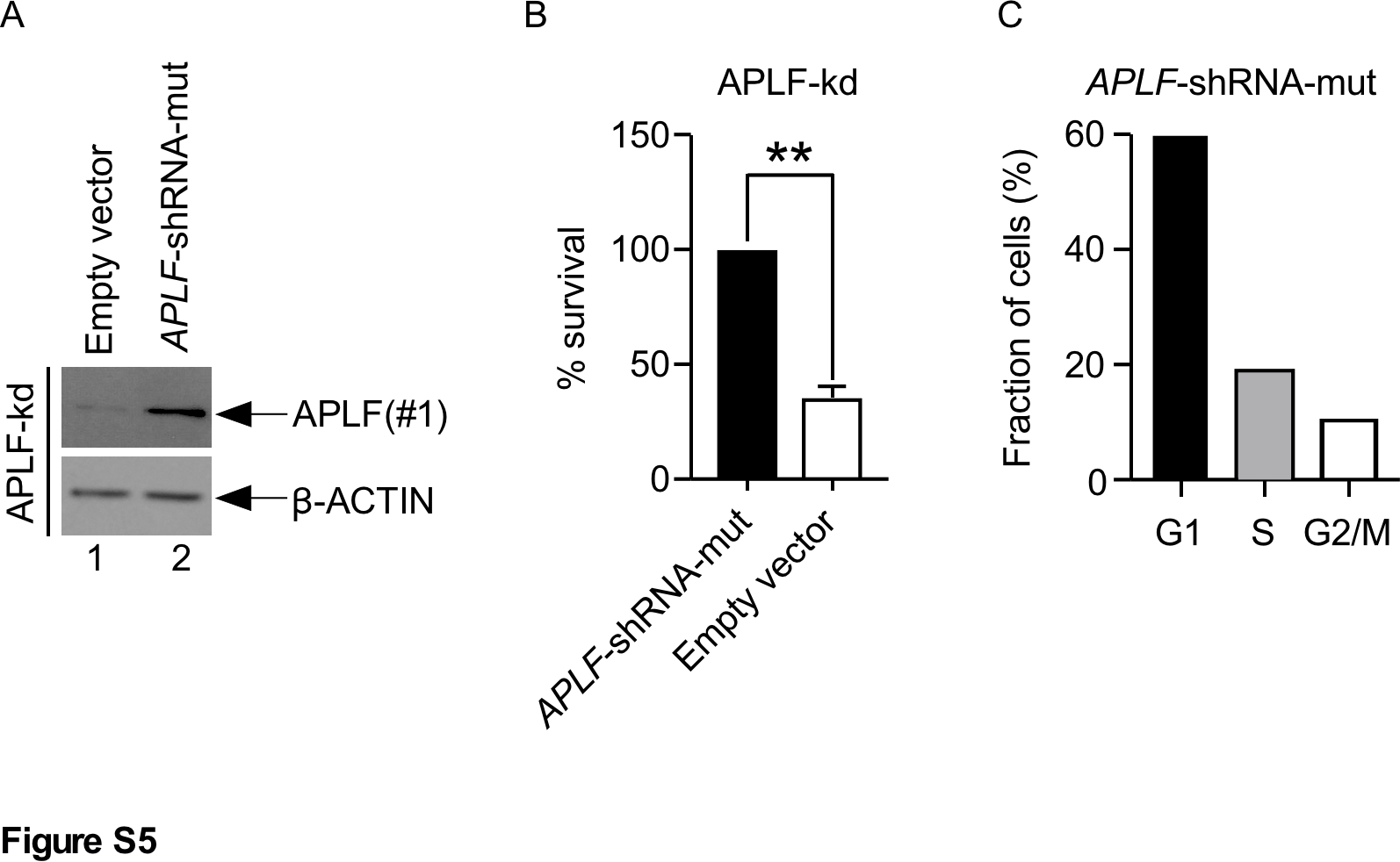

Supplement: Supplementary file 1 — Material & methods, Supplementary Figures, Tables. (ZIP 4889 kb) [file 12943_2018_826_MOESM1_ESM.zip › Figure S5.jpg]

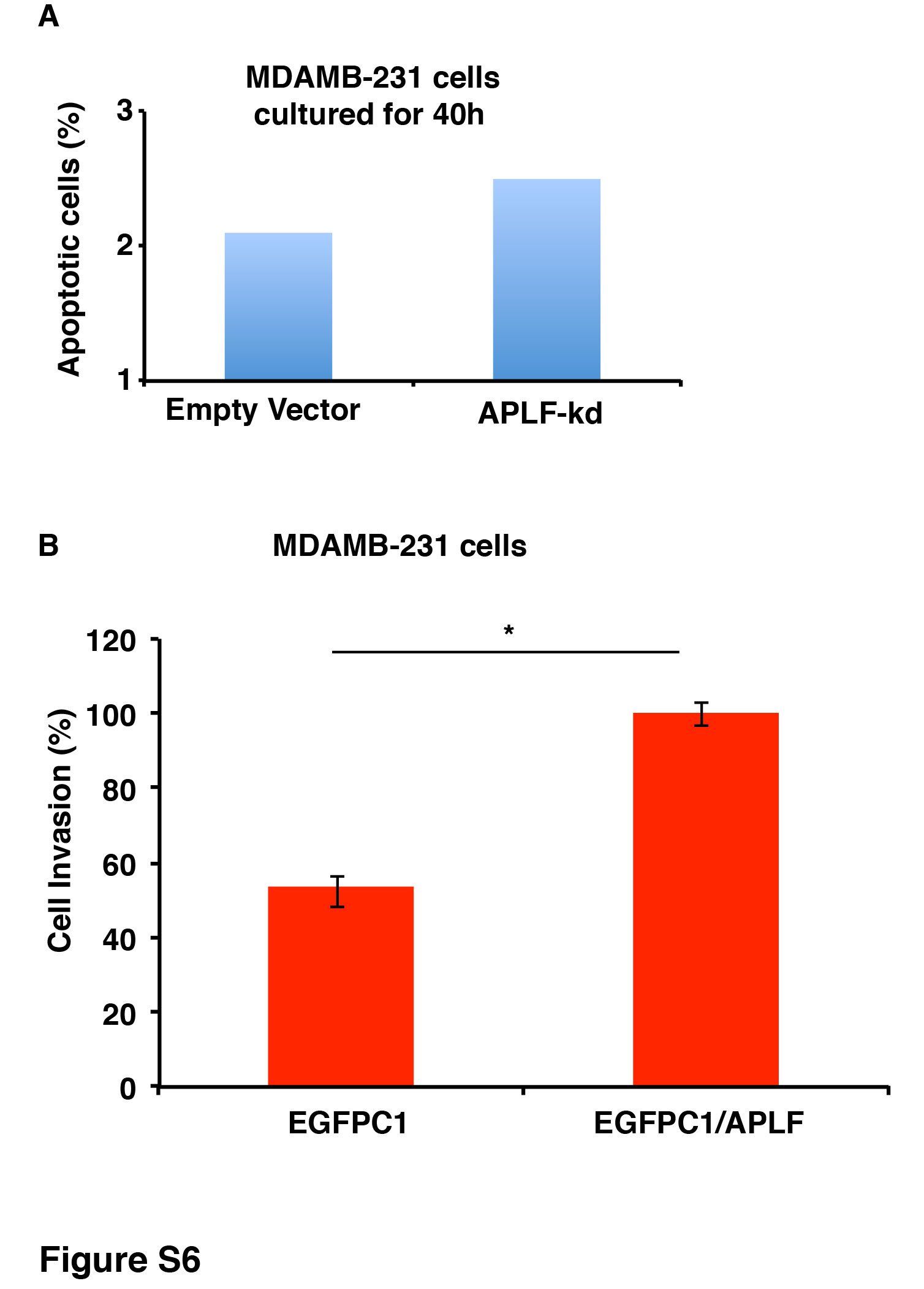

Supplement: Supplementary file 1 — Material & methods, Supplementary Figures, Tables. (ZIP 4889 kb) [file 12943_2018_826_MOESM1_ESM.zip › Figure S6.jpg]

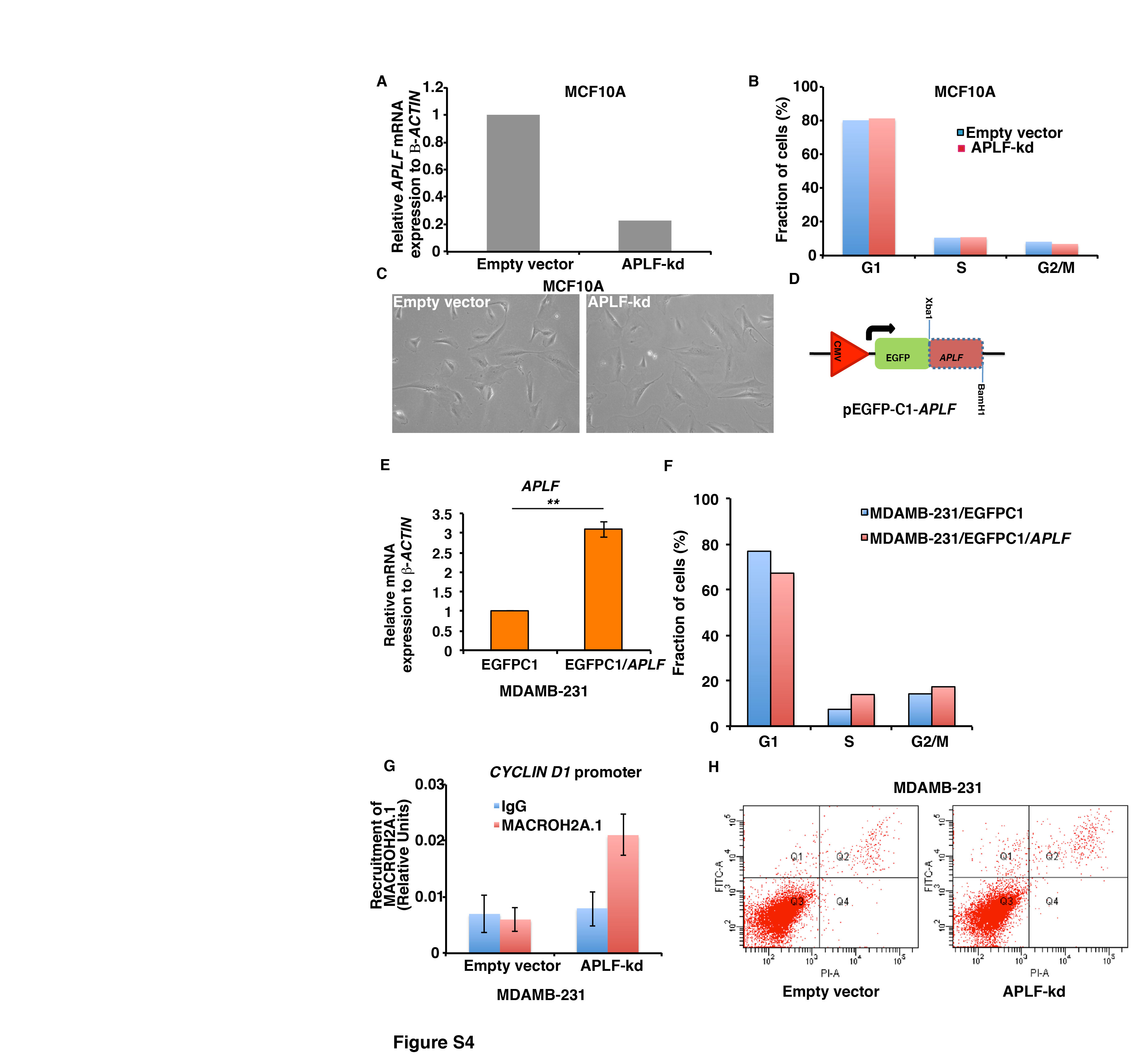

Supplement: Supplementary file 1 — Material & methods, Supplementary Figures, Tables. (ZIP 4889 kb) [file 12943_2018_826_MOESM1_ESM.zip › Figure S4.jpg]

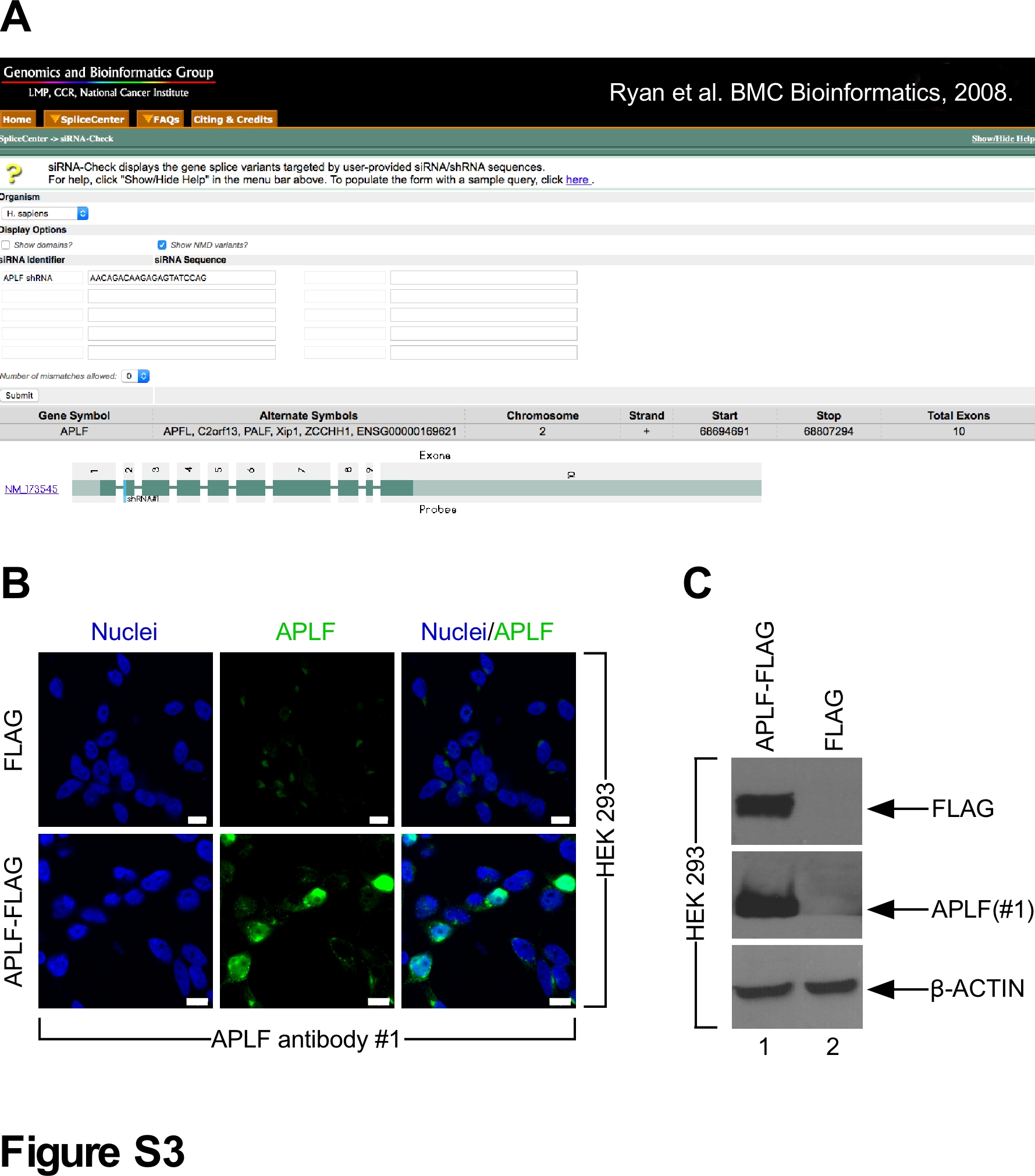

Supplement: Supplementary file 1 — Material & methods, Supplementary Figures, Tables. (ZIP 4889 kb) [file 12943_2018_826_MOESM1_ESM.zip › Figure S3.jpg]

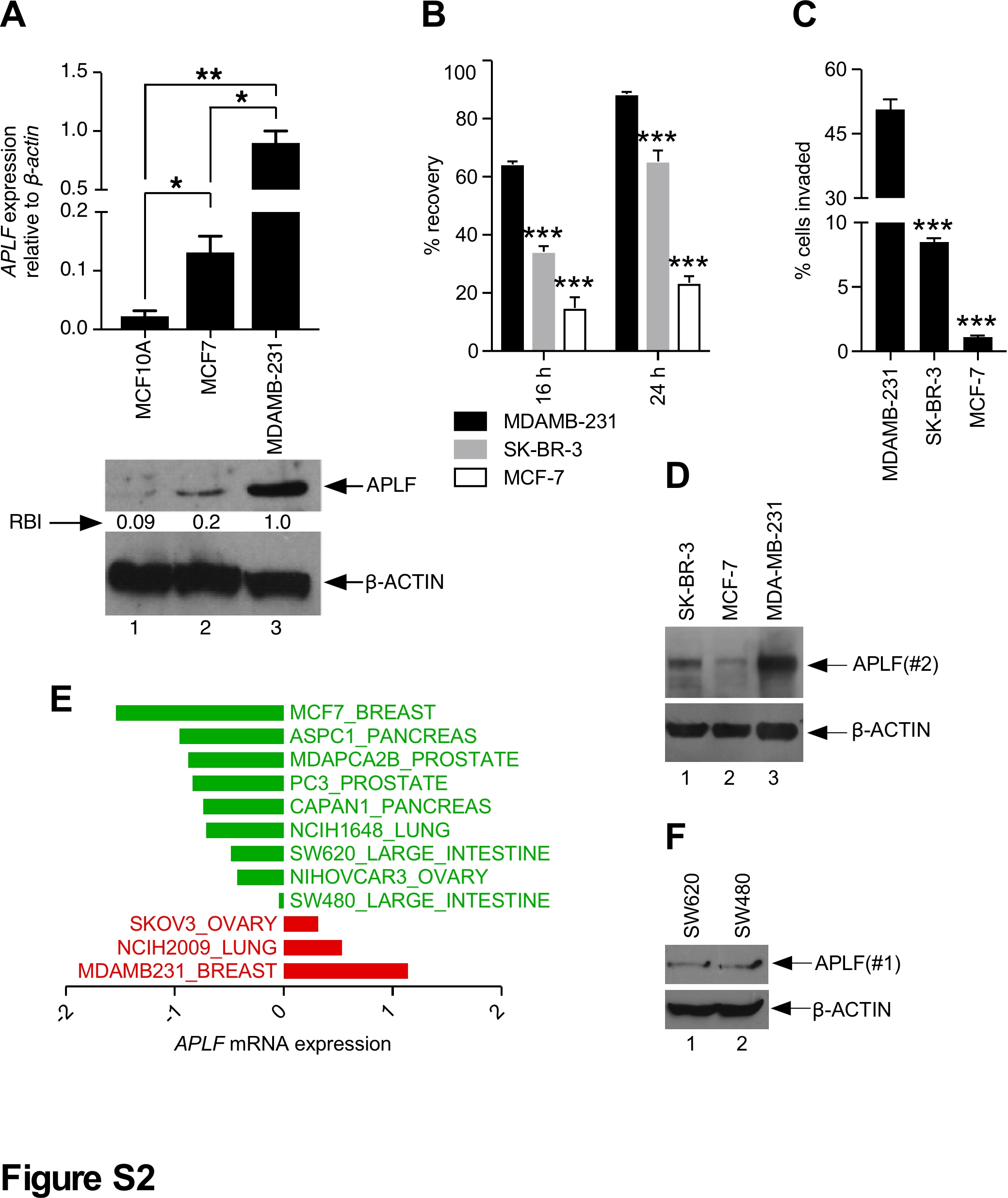

Supplement: Supplementary file 1 — Material & methods, Supplementary Figures, Tables. (ZIP 4889 kb) [file 12943_2018_826_MOESM1_ESM.zip › Figure S2.jpg]

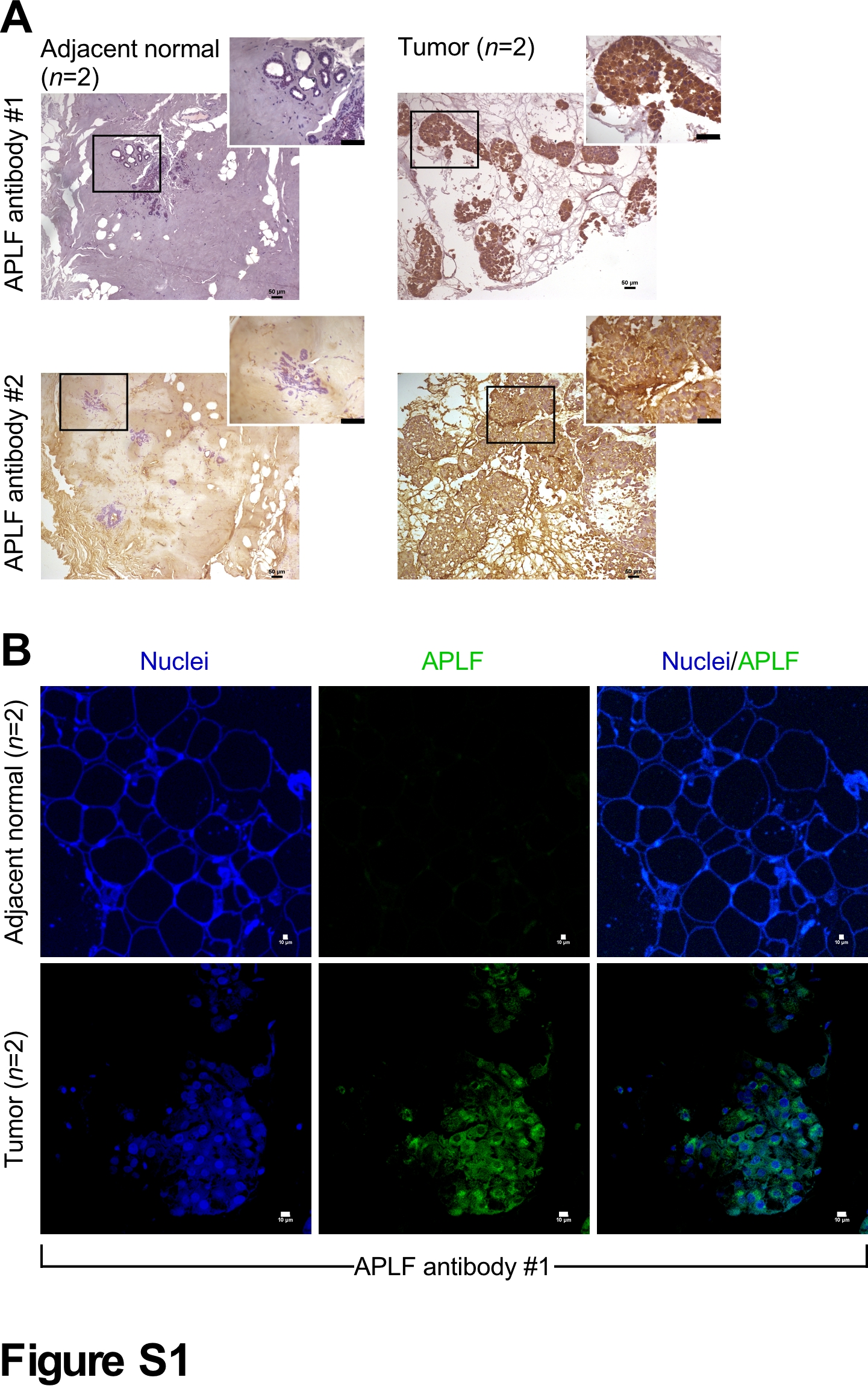

Supplement: Supplementary file 1 — Material & methods, Supplementary Figures, Tables. (ZIP 4889 kb) [file 12943_2018_826_MOESM1_ESM.zip › Figure S1.jpg]
